# Transcriptional profiling of long non-coding RNAs in mantle of *Crassostrea gigas* and their association with shell pigmentation

**DOI:** 10.1038/s41598-018-19950-6

**Published:** 2018-01-23

**Authors:** Dandan Feng, Qi Li, Hong Yu, Lingfeng Kong, Shaojun Du

**Affiliations:** 1 Key Laboratory of Mariculture, Ministry of Education, Ocean University of China, Qingdao, 266003 China; 2 Laboratory for Marine Fisheries Science and Food Production Processes, Qingdao National Laboratory for Marine Science and Technology, Qingdao, 266237 China; 3 Institute of Marine and Environmental Technology, Department of Biochemistry and Molecular Biology, University of Maryland School of Medicine, Baltimore, MD United States

## Abstract

Long non-coding RNAs (lncRNAs) play crucial roles in diverse biological processes and have drawn extensive attention in the past few years. However, lncRNAs remain poorly understood about expression and roles in *Crassostrea gigas*, a potential model organism for marine molluscan studies. Here, we systematically identified lncRNAs in the mantles of *C. gigas* from four full-sib families characterized by white, black, golden, and partially pigmented shell. Using poly(A)-independent and strand-specific RNA-seq, a total of 441,205,852 clean reads and 12,243 lncRNA transcripts were obtained. LncRNA transcripts were relatively short with few exons and low levels of expression in comparison to protein coding mRNA transcripts. A total of 427 lncRNAs and 349 mRNAs were identified to differentially express among six pairwise groups, mainly involving in biomineralization and pigmentation through functional enrichment. Furthermore, a total of 6 mRNAs and their *cis*-acting lncRNAs were predicted to involve in synthesis of melanin, carotenoid, tetrapyrrole, or ommochrome. Of them, chorion peroxidase and its *cis*-acting lincRNA TCONS_00951105 are implicated in playing an essential role in the melanin synthetic pathway. Our studies provided the first systematic characterization of lncRNAs catalog expressed in oyster mantle, which may facilitate understanding the molecular regulation of shell colour diversity and provide new insights into future selective breeding of *C. gigas* for aquaculture.

## Introduction

The large proportion of a eukaryotic genome is transcribed to produce a huge array of RNA molecules differing in protein-coding capability, size, and abundance^1^. Over the past decade, with the development of next-generation sequencing techniques, genome-wide transcriptome analysis, it was discovered that the genomes of eukaryotes encode a vast range of non-protein coding RNAs (ncRNAs)^2,3^. ncRNAs comprised of different types of small RNA (sRNA) and long noncoding RNAs (lncRNAs) that have been implicated in transcriptional and post-transcriptional regulation of gene expression or in guiding DNA modification^4^. Thousands of lncRNAs have been characterized in a limited number of eukaryotes. lncRNAs showed generally lower expression level, shorter length compared with counterpart mRNAs^2,3,5,6^. As expected for regulatory molecules, lncRNAs display specific spatiotemporal expression patterns, high tissue specificity and can regulate expression of genes in close genomic proximity (*cis*-acting) or at distance (*trans*-acting)^3,7^.

Many lncRNAs have been shown to play crucial roles in diverse biological processes^5,7^. Emerging evidence indicated that lncRNAs may have important roles in pigmentation. For example, the whole transcriptome analysis of pigmented and non-pigmented skin suggests a possible functional relevance of lncRNA in the modulation of pigmentation processes both in bovine^8^ and goat^9^. In goat, the impact of lncRNAs on its target genes in *cis* and *trans* was investigated, indicating that these lncRNAs have a strict tissue specificity and functional conservation^9^. Study on lncRNAs and their *cis*-target genes in melanocytes suggested the role in the melanogenesis^10^.

The fabulous and diverse colours of molluscan shells are generally believed to be determined by presence of biological pigments. The widely recognized shell colour diversities have been appreciated for hundreds of years by collectors and scientists alike^11^. However, characterization of the shell pigments and identification of molecular pathways involved in their synthesis in Mollusca lag behind the large numbers of studies undertaken on plants, vertebrates and insects^12–17^. At present, the main shell pigments found in Mollusca are carotenoids, melanin and tetrapyrroles, including porphyrins and bile pigments^11,18^. The molecular processes involved in the synthesis of pigment have been studied in only a few molluscs^19–24^. Of that, the regulatory mechanism for melanin synthesis is better known in cephalopods, involving in the activation of tyrosinase and increased melanin synthesis in the ink gland^25^. And it is noteworthy, some of the shell pigments have been shown to be produced via the highly conserved pathways. For instance, the tyrosinase enzyme, which plays extensive roles in eukaryotes, has been identified as the key enzyme in the pathway for melanin production in mollusks^26^.

The Pacific oyster, *Crassostrea gigas*, is a widely distributed and economically important species, belonging to Mollusca. Owing to its economical, biological and ecological importance, the biology and genetics of the Pacific oyster have been extensively studied, which enables *C. gigas* to be a potential model organism for marine mollusca studies^27^. Through successive family selection and breeding, we have developed four full-sib families characterized by shell colours (white, golden, black and partially pigmented). Digital gene expression profiling (DGE), which observed the abundance of a particular transcript as a count, discovered some differentially expressed genes and enriched pathways potentially involved in pigmentation, using those four shell colour variants^20^. The recently released genome sequence of *C. gigas* enabled us to develop a pipeline to identify 11,668 long intergenic non-coding RNAs (lincRNAs) from different tissues and developmental stages, based on RNA-seq resources available^2^. However, the whole lncRNAs catalog of *C. gigas* is not well characterized in any tissue, let alone their association with pigmentation.

In this study, we compiled the first genome-wide catalog of lncRNAs in mantle of *C. gigas* characterized by shell colour using poly(A)-independent and strand-specific RNA-seq. This comprehensive database of lncRNAs could serve a valuable framework that can be applied to further large-scale lncRNAs screen in *C. gigas*. Our study provides a valuable resource for studying lncRNAs in mantle of *C. gigas*, as well as contributes to better understanding the shell pigmentation.

## Materials and Methods

### Sample collection and preparation

Four kinds of *C. gigas* lines of full-sib families, named as the white shell (WS), black shell (BS), golden shell (GS), and normal or partially pigmented shell (NS) full-sib families were established. These families were developed by six-generation successive family selection and exhibited steadily hereditary shell colour traits. The original parents of white, black, golden and normal *C. gigas* were selected from locally cultured populations in Weihai, Shandong, China. In 2015, we respectively sampled six oyster individuals of five-month-old from four full-sib families for RNA-seq. Left mantle was dissected and stored in RNA store (Dongsheng Biotech) before RNA extraction. Four mantle samples, respectively named the black shell oyster mantle (BSM), the golden shell oyster mantle (GSM), normal or partially pigmented shell oyster mantle (NSM), and the white shell oyster mantle (WSM), were used for RNA-seq.

### RNA isolation, library preparation and sequencing

The mantle from each individual was lysed in 1 ml of Trizol Reagent (Invitrogen, Carlsbad, CA) for total RNA extraction according to the manufacturer’s instructions. RNA quality and contamination was checked on 1% agarose gels. RNA purity, concentration, integrity were checked using the NanoPhotometer® spectrophotometer (IMPLEN, Westlake Village, CA), Qubit® RNA Assay Kit in Qubit® 2.0 Flurometer (Life Technologies, Carlsbad, CA), and the RNA Nano 6000 Assay Kit of the Bioanalyzer 2100 system (Agilent Technologies, Santa Clara, CA), respectively.

At least 3 µg of total RNA was pooled proportionally from six individuals within each family, a total of four samples were used for library construction. Firstly, ribosomal RNA was removed by Epicentre Ribo-zero^TM^ rRNA Removal Kit (Epicentre, Madison, WI), and rRNA free RNA sample was cleaned up by ethanol precipitation. Subsequently, sequencing libraries were generated using the rRNA-depleted RNA by NEBNext^®^ Ultra^TM^ Directional RNA Library Prep Kit for Illumina® (NEB, Ipswich, MA). In order to select cDNA fragments of preferentially 150~200 bp in length, the library fragments were purified with AMPure XP system (Beckman Coulter, Beverly, MA). Finally, the library quality was assessed on the Agilent Bioanalyzer 2100 system. The clustering of the index-coded samples was performed on a cBot Cluster Generation System using the TruSeq PE Cluster Kit v3-cBot-HS (Illumina). After cluster generation, the libraries were sequenced on an Illumina Hiseq. 4000 of the Novogene Bioinformatics Institute (Beijing, China), and 150 bp paired-end reads were generated.

### Quality control and transcriptome assembly

The RNAseq data were cleaned by removing reads containing adapter or poly-N and low quality reads from raw data. A total of 441,205,852 clean reads were obtained after quality filter from a total of 465,803,034 raw 150-bp paired-end reads. And the downstream analyses were based on the clean data with high quality. Q20, Q30, and GC contents of the clean data were calculated. The complete dataset was deposited into NCBI’s Sequence Read Archive (PRJNA381520/SUB2554964).

Reference genome and gene model annotation files were downloaded from genome website (ftp://ftp.ncbi.nlm.nih.gov/genomes/Crassostrea_gigas). Index of the reference genome was built using Bowtie v2.0.6 and paired-end clean reads were aligned to the reference genome using TopHat v2.0.9. The mapped reads of each sample were assembled by Cufflinks (v2.1.1) in a reference-based approach^28^.

### Identification and characterization of lncRNAs

We developed a stringent filtering pipeline designed to remove transcripts with evidence for protein-coding potential based on current approaches: (i) Filter out single-exon transcripts nearest distance ≤500 bp with other transcripts, which might be extended exons of annotated protein-coding genes^2^. (ii) Remove transcripts with short lengths (<200 bp). (iii) Select single-exon transcripts with FPKM ≥ 2 and multiple-exon transcripts with FPKM ≥ 0.5. (iv) Filter out transcripts that belong to tRNA, rRNA, snoRNA, snRNA, pre-miRNA, and pseudogenes by cuffcompare. (v) The remaining transcripts were blasted with known mRNA and completed the preliminary screening. (vi) Classify candidate lncRNAs into three subtypes (lincRNA, intronic lncRNA, and antisense lncRNA) using information of calss_code of cuffcompare. (vii) The tools of CPC (Coding Potential Calculator), CPAT (Coding-Potential Assessment Tool), Pfamscan were used to detect putative protein encoding transcripts, potential lncRNA transcripts were retained which are not detected in any tool. (viii) Select putative lncRNAs which can be detected in at least three libraries.

### Characterization and quantification of transcripts

RepeatMasker (http://www.repeatmasker.org) was used with default parameters to identify various TE components in oyster.

*Cis* role is lncRNA acting on neighboring target genes. For the *cis* action of lncRNAs, we searched for protein-coding genes 100 kb upstream and downstream of the lncRNAs, respectively. Cuffdiff (v2.1.1) was used to calculate FPKMs of both lncRNAs and coding genes transcripts in each sample^28^. Transcripts with *P*-adjust < 0.05 and the absolute value of log2 (Fold change) >1 were described as differentially expressed between any two shell colours, which were profiled as differentially expressed transcripts (DETs). Differentially expressed mRNA assemblies (DEM)^20^ were also independently analyzed and recorded in six pairwise groups, which were detected from the same four shell colours oyster lines. Shared differentially expressed genes from the same two pairwise groups were retained.

### Functional enrichment analysis

Gene Ontology (GO) enrichment analysis of differentially expressed genes or lncRNA target genes was implemented by the GOseq R package, in which gene length bias was corrected. GO terms with corrected *P* value less than 0.05 were considered significantly enriched by differential expressed genes.

KEGG is a database resource for understanding high-level functions and utilities of the biological system. We used KOBAS software to test the statistical enrichment of differentially expressed genes or lncRNA target genes in KEGG pathways. Hypergeometric *P* value < 0.05 was considered significant.

### Validation of gene expression by quantitative PCR analysis

To validate the RNA-seq data, 16 differentially expressed transcripts of interest were selected for quantitative real-time PCR (qPCR) analysis. Total RNA was extracted separately from the same 24 samples used for RNA sequencing. Then cDNA was synthesized from RNA, which was used for qPCR, using Prime Script TM RT reagent Kit with gDNA Eraser (TaKaRa, Dalian, China). Specific primers for qPCR were designed using Premier Primer 5 (Supplementary Table S1) and verified by NCBI primer-BLAST. Elongation Factor was used as an endogenous control^29^. The amplification was performed on the LightCycler 480 real-time PCR instrument (Roche Diagnostics, Burgess Hill, UK) using SYBR® Premix Ex TaqTM (TaKaRa). Cycling parameters were 95 °C for 5 min, then 40 cycles of 95 °C for 5 s, 60 °C for 20 s. Melting curve analyses were performed following amplifications to verify specific amplication. Relative gene expression data was analyzed using the comparative threshold cycle (CT) method^30^. Data were examined for homogeneity of variances (F text), and were analyzed by t test using software SPSS 13.0 with *P* < 0.05. Eight oysters, typically having black for the left shell and white for the right shell, were also picked up for qPCR.

## Results

### Identification and characterization of lncRNAs in oyster mantles

**Figure 1:**
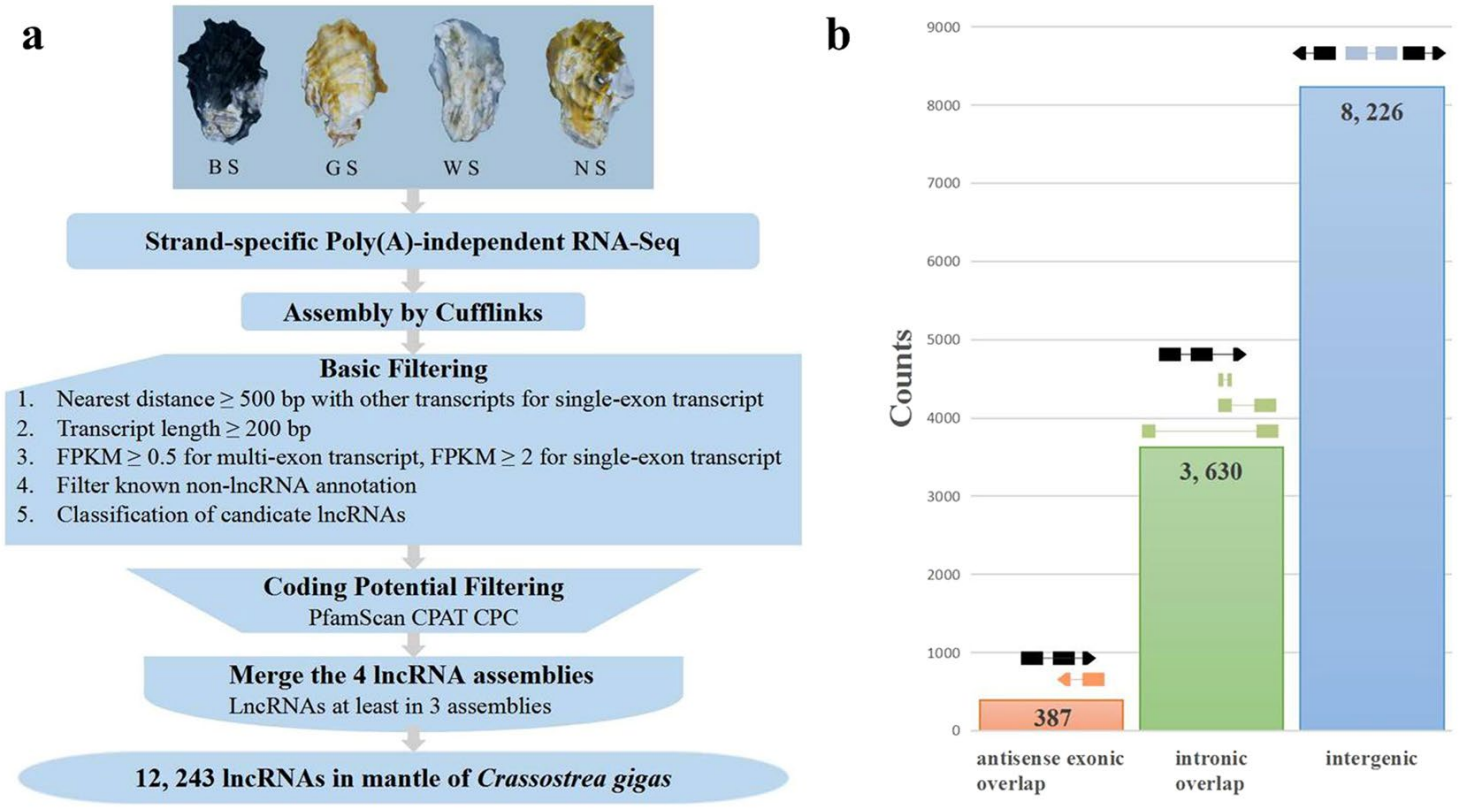
Identification and classification of *Crassostrea gigas* lncRNAs. (**a**) Overview of the computational filtering pipeline used for the identification of oyster lncRNAs. See main text and Materials and Methods for details. Ellipse box highlights the final number of transcripts that passed all filters and were considered high-confidence oyster lncRNAs. (**b**) Number of lncRNAs in each of the three main classes defined by their genomic location relative to protein-coding genes. A schematic representation of lncRNAs (colour) position relative to protein-coding genes (black) is shown on the top. lncRNAs with “antisense exonic overlap” (red) have at least one exon that overlaps with an exon of a protein-coding gene on the opposite strand. lncRNAs with “intronic overlap” (green) are defined as transcripts that have overlap with another protein-coding gene but no exon–exon overlap (no overlap with exons of the overlapping genes). “Intergenic” lncRNAs (blue) have no overlap with any protein-coding gene.

The number of RNA-seq reads, quality of the reads, and the mapping rate for each of the four libraries sequenced are summarized (Table 1). A total of 99,092 transcripts were assembled by the Cufflinks, which were used for subsequent analysis. Using the criteria shown in Fig. 1a, 12,243 lncRNAs transcripts expressed in mantle were identified from at least three of the four samples analyzed. They consist of 8,226 lincRNAs, 387 antisense lncRNAs, and 3,630 intronic lncRNAs (Fig. 1b). These lncRNA transcripts correspond to 11,637 lncRNA gene loci. In addition, 45,393 protein-coding transcripts were also identified.

**Table 1:** Basic characteristic of reads in four libraries and data of sequencing reads mapping to the reference genome.

**Sample name**	**BSM**	**GSM**	**NSM**	**WSM**
Raw reads	99,758,320	109,996,464	119,299,572	136,748,678
Clean reads	93,987,492	103,305,608	112,944,804	130,967,948
Clean bases	14.1 G	15.5 G	16.94 G	19.65 G
Error rate(%)	0.02	0.01	0.02	0.01
Q20(%)	96.91	97.1	96.96	97.6
Q30(%)	92.47	92.81	92.57	93.88
GC content(%)	44.08	46.57	44.08	45.16
Total mapped	70571864 (75.09%)	81660962 (79.05%)	84586510 (74.89%)	100807144 (76.97%)
Multiple mapped	7312694 (7.78%)	9367467 (9.07%)	7700828 (6.82%)	10709954 (8.18%)
Uniquely mapped	63259170 (67.31%)	72293495 (69.98%)	76885682 (68.07%)	90097190 (68.79%)
Read-1	32615939 (34.7%)	37203463 (36.01%)	39517852 (34.99%)	45957811 (35.09%)
Read-2	30643231 (32.6%)	35090032 (33.97%)	37367830 (33.09%)	44139379 (33.7%)
Reads map to ‘+’	31768580 (33.8%)	36272547 (35.11%)	38639356 (34.21%)	45154870 (34.48%)
Reads map to ‘−’	31490590 (33.51%)	36020948 (34.87%)	38246326 (33.86%)	44942320 (34.32%)
Non-splice reads	41421851 (44.07%)	50059318 (48.46%)	52748486 (46.7%)	58198638 (44.44%)
Splice reads	21837319 (23.23%)	22234177 (21.52%)	24137196 (21.37%)	31898552 (24.36%)

**Figure 2:**
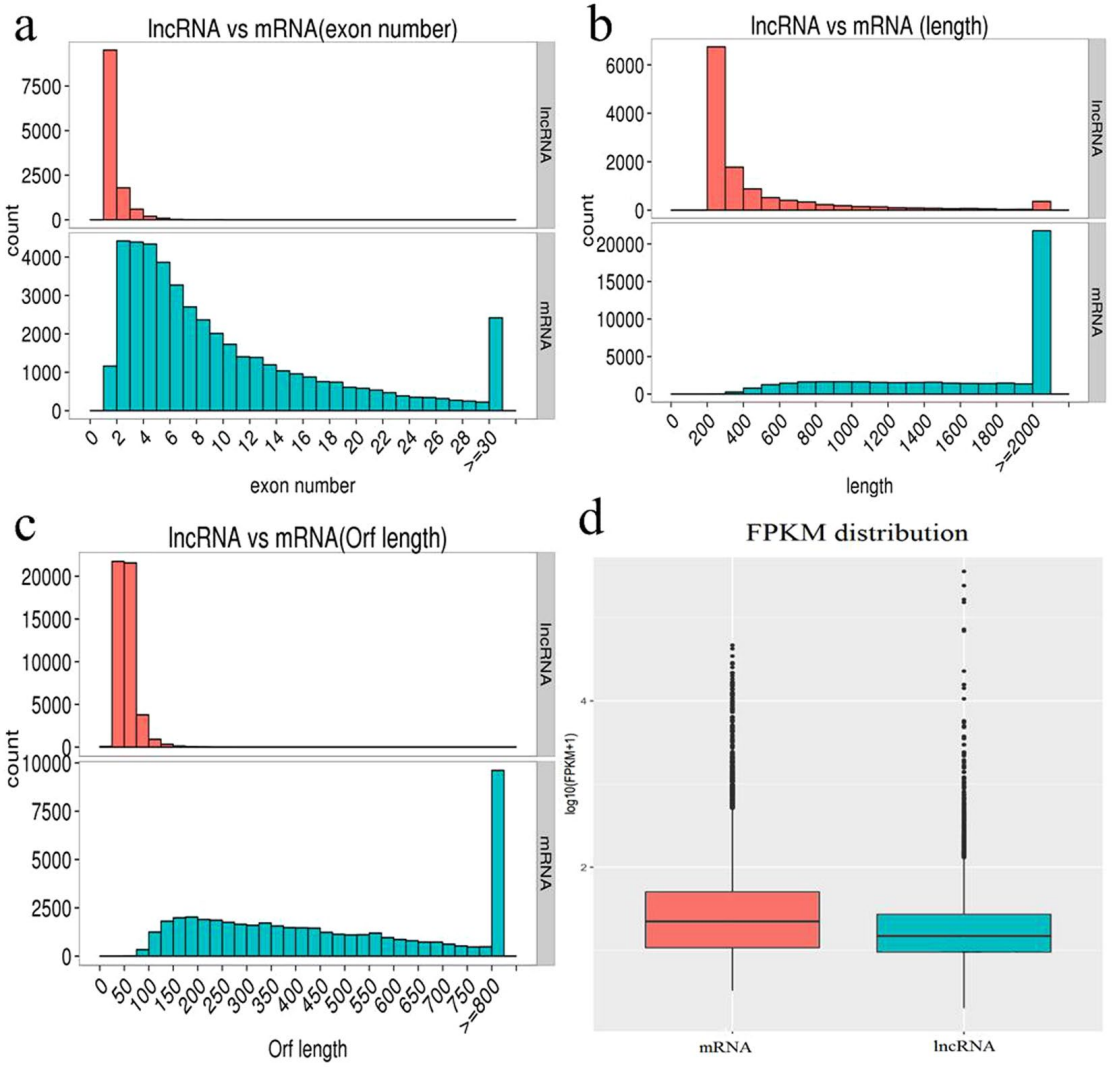
Comparison of the identified lncRNAs and mRNAs. (**a**) Distribution of the number of exons in the mRNAs and lncRNAs. (**b**) Distribution of transcript lengths in the mRNAs and lncRNAs. (**c**) Distribution of open reading frame lengths in the mRNAs and lncRNAs. (**d**) Expression level analysis in the mRNAs and lncRNAs.

The exon number, sequence length, open reading frame length, and expression levels were characterized for the obtained 12,243 lncRNAs and 45,393 mRNAs. Our results indicated that most of lncRNAs contained fewer exons (one or two) than mRNAs (Fig. 2a). The distribution of transcript length was obviously different. The average length of lncRNAs was shorter than that of mRNAs (Fig. 2b), and the lncRNAs in our dataset tend to be shorter in open reading frame length than mRNAs (Fig. 2c). In addition, lncRNAs exhibited a lower level of expression than mRNA (Fig. 2d). With the absence of lncRNAs data in Mollusca, we failed to properly evaluate the conservation of lncRNAs in the oyster.

Furthermore, we identified differences in Transposable element (TE) components between lncRNAs and mRNAs, as well as among the three lncRNA subtypes. Our analysis revealed TE component characteristics that distinguished the four transcripts subtypes (Fig. S1; Supplementary Table S2). At the global level, Class II DNA transposons, minisatellite, and rollingcircle helitron (RC/Helitron) were the three most abundant known repetitive elements to overlap with oyster transcripts. Significant differences in the percentage of TE components were observed between mRNAs and the individual subtype of lncRNAs. A total of 23, 396 TEs were found in 8, 281 lncRNAs, which account for 67.64% (8, 281/12, 243) of the total number of lncRNAs. A total of 521, 392 TEs were found in 40, 120 mRNAs, which account for 88.38% (40, 120/521, 390) of the total number of mRNAs. The results revealed that TE percentage in oyster is considerably lower for lncRNAs (67.64%) than for protein-coding genes (88.38%).

### Analysis of differentially expressed transcripts

The expression levels of lncRNA and mRNA transcripts were estimated by fragments per kilobase per million fragments mapped (FPKM). The differentially expressed transcripts were detected between any two samples. As a result, a total of 427 differentially expressed lncRNA transcripts were identified among six pairwise groups, of which 183 lncRNA transcripts were differentially expressed in black oyster relative to white oyster (Supplementary Tables S3 and S4). The 427 differentially expressed lncRNA transcripts corresponded to 411 lncRNA gene loci. Cluster analysis of differentially expressed lncRNAs was revealed by a heat map, which showed four samples clustered separately (Fig. S2a).

**Figure 3:**
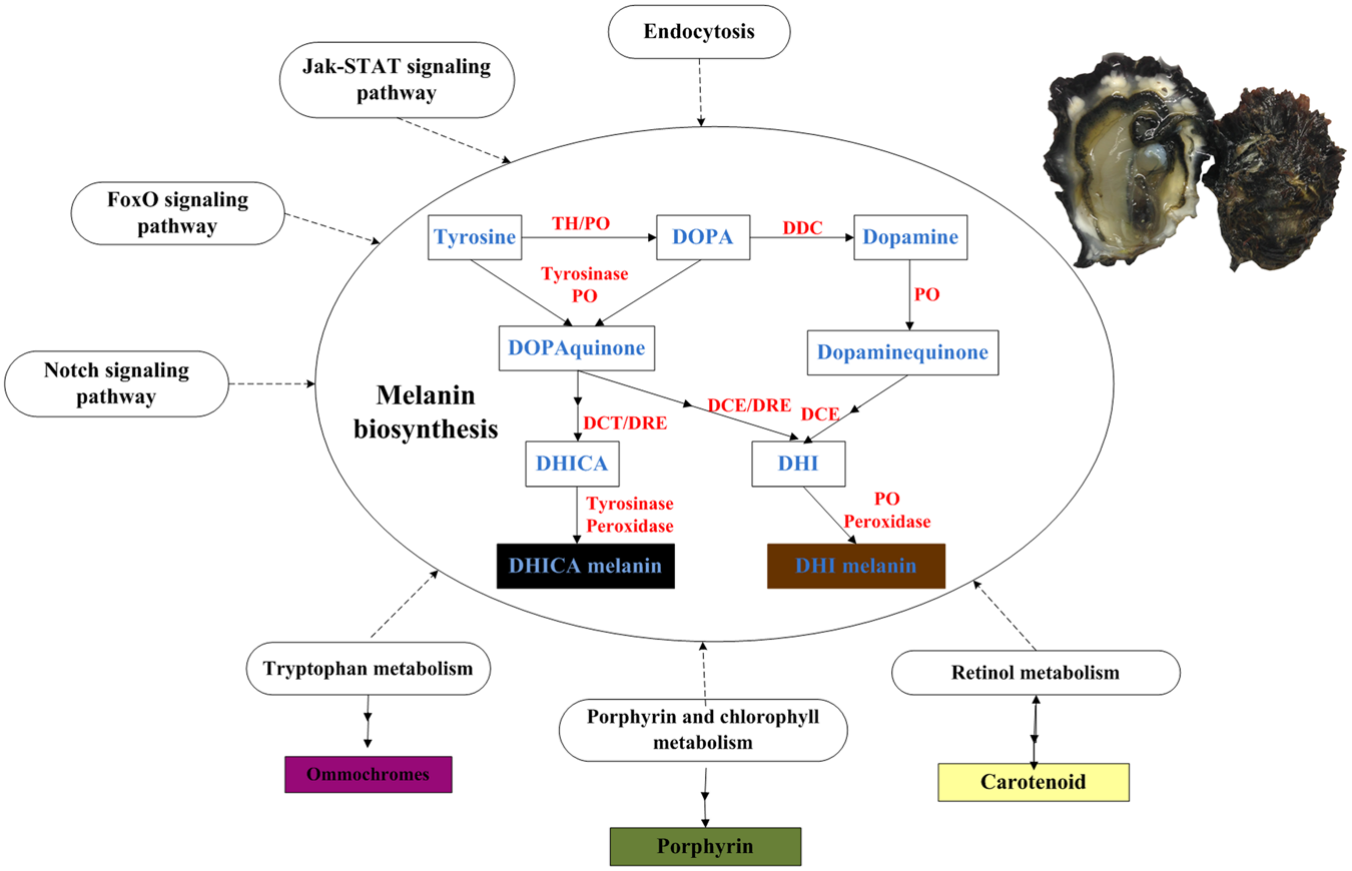
Common pathways of melanin biosynthesis in animals and its related pathways. Enzymes are abbreviated as follows: tyrosine hydroxylase (TH), phenoloxidase (PO), DOPA decarboxylase (DDC), dopachrome tautomerase (DCT), dopachrome conversion enzyme (DCE), dopachrome rearranging enzymes (DRE). Pigment precursors are shown in blue, enzyme are shown in red. Some signaling pathways are indicated around melanin biosynthesis, which were significantly identified in this study and reported to regulate melanin biosynthesis in animals. Some metabolism pathways are also indicated around melanin biosynthesis, which are significantly identified here and reported to involve in other pigments biosynthesis.

A total of 1, 289 differentially expressed mRNAs (DEMs) were identified among six pairwise groups using cutoff of log_2_ (fold_change) >1 and *q*-value < 0.05 (Supplementary Tables S3 and S5), DEMs in these four samples showed the consistent cluster pattern with differentially expressed lncRNA transcripts (Fig. S2b). By integrating two DEMs assemblies from six pairwise comparison groups of four different shell colour oyster families, there were 94, 79, 140, 53, 118, and 79 DEMs were identified, respectively (Supplementary Tables S3 and S5), resulting in a total of 349 significantly DEMs (Supplementary Tables S3 and S6). A total of 6 mRNAs were confirmed by qPCR (Supplementary Table S1). These 6 genes are known to play essential roles in pigment biosynthesis of melanin, tetrapyrrole, carotenoid, and ommochrome (Table 2; Fig. 3).

**Table 2:** LncRNAs and their potential *cis*-acting genes that are involved in pigmentation.

**Gene_id**	**Gene description from** ***Swiss prot***	**Related pigments**	**LncRNAs in** ***cis*** **-acting**			**GO_molecular_function_description**	
LOC105344040	Tyrosinase-like protein 2	melanin	TCONS_00117832	TCONS_00117563	TCONS_00117745	GO:0016491	oxidoreductase activity
			TCONS_00117906				
LOC105324831	Tyrosinase-like protein 3	melanin	TCONS_00821172	TCONS_00820679	TCONS_00820814	GO:0016491	oxidoreductase activity
			TCONS_00820688	TCONS_00820896			
LOC105334556	Dopamine	melanin	TCONS_00909452	TCONS_00909160	TCONS_00909168	GO:0016491	oxidoreductase activity
	beta-monooxygenase		TCONS_00907826	TCONS_00909199			
LOC105324712	Chorion peroxidase	melanin, tetrapyrrole	TCONS_00951105	TCONS_00950402	TCONS_00950769	GO:0046906//GO:0016491	tetrapyrrole binding//oxidoreductase activity
LOC105336634	Cytochrome P450 2U1	carotenoid, melanin	TCONS_00454937	TCONS_00454715	TCONS_00454720	GO:0046906///GO:0016491	tetrapyrrole binding//oxidoreductase activity
		tetrapyrrole,	TCONS_00455648				
LOC105326901	Kynurenine 3-	melanin	TCONS_00119630	TCONS_00120204	TCONS_00119485	GO:0016491	oxidoreductase activity
	Monooxygenase	ommochrome,	TCONS_00120197	TCONS_00119738

**Figure 4:**
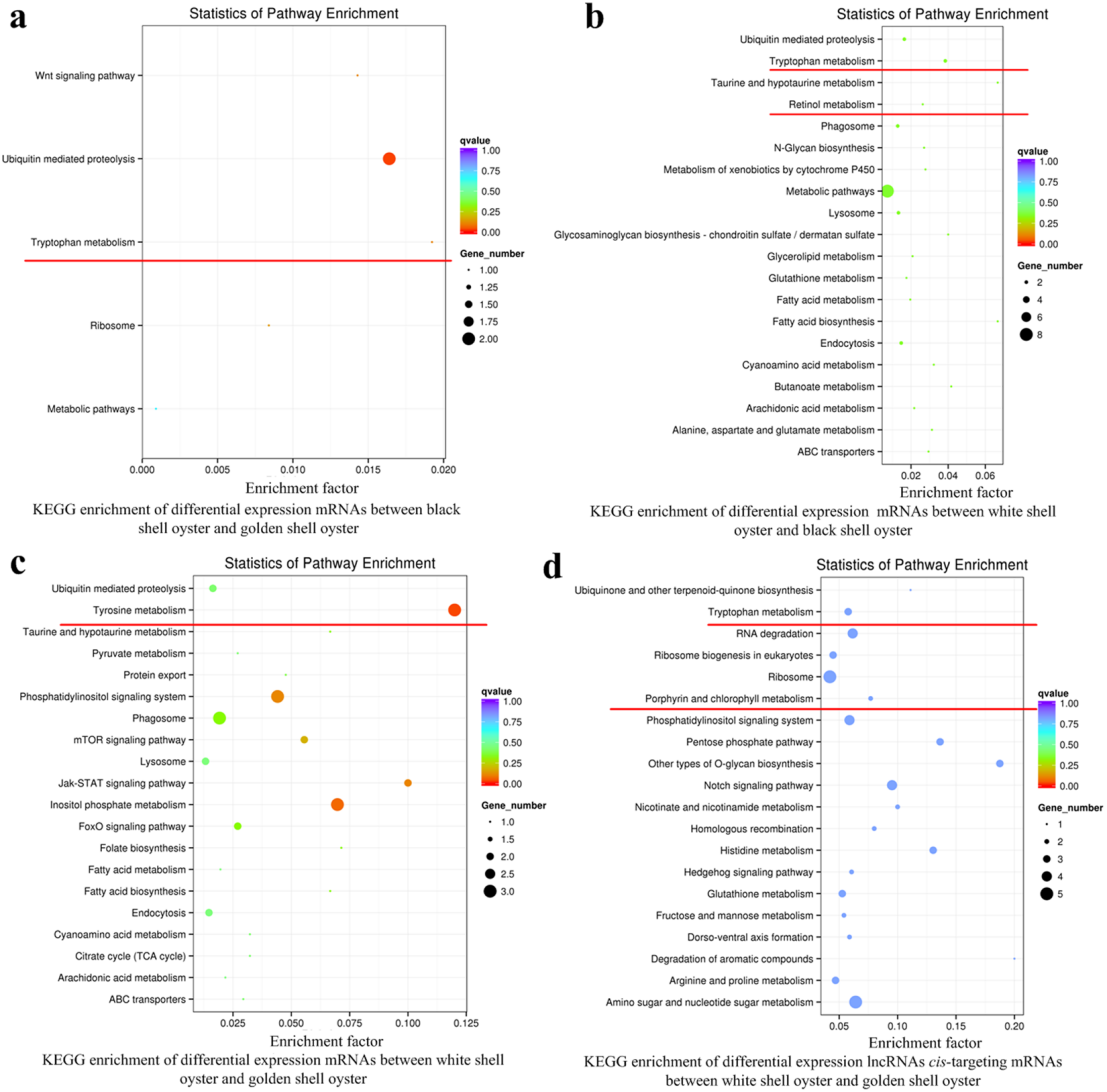
KEGG enrichment analysis of differentially expressed transcripts. The *y*-axis represented the KEGG enriched pathways, the *x*-axis represented the enrichment factor, which was calculated by ratio of the number of differentially expressed transcripts divided by the number of annotated transcripts in this pathway. The potential pigmentation-related pathways were underlined by red line.

GO and KEGG analyses were also performed on 349 significantly differentially expressed mRNA. As the results, we derived 13 highly enriched GO terms (Supplementary Table S7) and 10 significantly enriched KEGG pathways (Supplementary Table S8). Importantly, we also observed several pigment biosynthesis related terms, such as “tyrosine metabolism”, “tryptophan metabolism”, and “retinol metabolism” in the data from KEGG analyses (Fig. 4).

Among the significantly differentially expressed lncRNA and mRNA transcripts, a total of 16 transcripts, including 6 lncRNAs and 10 mRNAs, were selected to confirm the utility of RNA-seq for quantitative analyses using quantitative polymerase chain reaction (qPCR). The results show that these transcripts were differentially expressed among different shell colours oysters and generally exhibited consistent with RNA sequencing data (Supplementary Table S1). Using oysters of asymmetric shell pigment pattern, we found that peroxidase, TCON_00924022, and TCON_00951105 showed a higher expression level in left mantle representing black shell than in right mantle representing white shell. Other six transcripts showed no significantly difference.

### The *cis* role of lncRNAs in target genes

To investigate the function of lncRNAs, we performed bioinformatics analysis identifying the potential targets of lncRNAs in *cis*. Our analysis included 11,157 lncRNAs that are associated with 24, 057 protein-coding genes within a range of 100 kb. We identified 427 differentially expressed lncRNA genes potentially targeting to 2,088 protein coding genes. GO analysis of the lncRNA *cis*-acting mRNA targets revealed two over-represented terms including RNA methyltransferase activity and tRNA methyltransferase activity that are primarily involved in regulation of gene expression (Supplementary Table S7). Pathway analysis showed that these lncRNAs *cis*-acting target genes were mainly enriched in six KEGG pathways involved in ECM-receptor interaction, Ubiquitin-mediated proteolysis, Jak-STAT signaling pathway, Notch signaling pathway, Homologous recombination and other types of O-glycan biosynthesis (Supplementary Table S8).

Through GO survey using lncRNAs *cis*-acting target genes in six pairwise groups, only one GO term of “Cysteine-type endopeptidase inhibitor” was significantly enriched when comparing BS with WS (Supplementary Table S7). In addition, 16 enriched Kyoto Encyclopedia of Genes and Genomes (KEGG) pathways were detected (Supplementary Table S8). Of these pathways, four pathways have been reported to be implicated in biomineralization^31–33^, including ECM-receptor interaction, Other types of O-glycan biosynthesis, Ubiquitin mediated proteolysis, and Pantothenate and CoA biosynthesis. Five pathways have been previously reported to regulate pigmentation, including the Jak-STAT signaling pathway^34,35^, Endocytosis^20,36^, FoxO signaling pathway^37,38^, and Notch signaling pathway^22^ (Fig. 3). It is noteworthy that several pigment biosynthesis related terms, such as “Tryptophan metabolism”, and “Porphyrin and chlorophy II metabolism” were also identified (Figs 3 and 4). Taken together, data from these functional enrichment analyses showed that these *cis*-acting genes of lncRNAs mainly involved in regulation of biomineralization and pigmentation.

### Association study

To further ascertain that lncRNA-protein-coding gene pairs exhibited DNA co-localization (for *cis*-acting) and expression correlation relationships, detailed examination was conducted. To deepen our understanding of the relationship between lncRNAs and pigmentation, first, we selectively analyzed pairs, in which the lncRNAs and their target genes were significantly differentially expressed between any two shell colours (Table S9). At the same time, gene annotation was used to identify the lncRNA-protein-coding gene pairs associated with pigment biosynthesis. According to these selective criteria, we found that chorion peroxidase, a potential pigment synthesis gene, and its *cis*-acting lincRNA TCONS_00951105 were detected to higher expression levels in pigmented oysters compared to white shell oysters, which were also confirmed in asymmetric oysters (Supplementary Table S1). We predicted that this lincRNA was probably involved in shell pigmentation. However, uncovering the definitive function of the predicted lncRNA requires additional verification studies.

## Discussion

In this study, we represented the first long non-coding transcripts catalog expressed in *C. gigas* mantle and analyzed their association with shell pigmentation. Our study not only enriched the knowledge of lncRNAs in marine invertebrate, but also provided new insights into potential functions of lncRNAs in molluscs. These RNA-seq data might provide molecular targets assisting the selective breeding of *C. gigas*.

These lncRNAs share many characteristics of their eukaryotic counterparts: such as shorter length, fewer exons, lower levels of expression compared with mRNAs. Conservation is missing because the lncRNA catalog in other molluscs is unaccessible. A previous study has estimated the relatively low conservation of lincRNA in *C. gigas*^2^. The same characteristics were also detected in lncRNAs found in sponge, goat, and other eukaryotes^1,3,9^. These common factors of lncRNAs in eukaryotes perhaps indicate their essential regulation during development. In addition to the preliminary examination of lncRNAs, we performed an extensive characterization to reveal major differences in transposon element (TE) components among mRNAs, lincRNAs, intronic lncRNAs, and anti-sense lncRNAs, which may be responsible for the observed differences in their evolution and function. TEs are mobile genetic elements that are capable of movement and proliferation within the genome. TEs are also considered as one of three evolutionary scenarios involved in the origin of lncRNAs^39^. TE coverage in *C. gigas* is considerably lower for lncRNAs than for protein-coding mRNAs. Thus, although little is known about repetitive elements in oyster, our findings are consistent with TEs being the origin of protein-coding genes than lncRNAs in *C. gigas*, which has also been proposed in *Amphimedon*^3^.

Unlike mRNA sequences that could provide potential information regarding their function, the sequence motifs of lncRNA are usually uninformative for predicting lncRNA function given that lncRNA functions are highly complex and diverse^40^. We predicted the potential function of lncRNAs in oyster mantle by analyzing their *cis*-acting protein-coding gene targets. Although this may not be the most appropriate model to explain the function of lncRNAs, GO analysis of all differentially expressed lncRNA *cis*-acting mRNAs identified two overrepresented terms of RNA methyltransferase activity and tRNA methyltransferase activity. RNA methylation has been reported to play a vital role in post-transcriptional regulation of gene expression^41,42^. Our studies were focused on the characterization of differentially expressed lncRNAs and their *cis*-acting mRNAs, and uncovering their potential functions by GO and KEGG analyses.

It has been reported in mollusk that shell colour is regulated by shell matrix proteins (SMPs) expressed in different shell layers^11^. While some of these proteins may have a role in shell colour determination, it is possible that these genes may play other roles in shell construction^24^. Notably, GO functional annotation analysis showed that only one GO term, namely Cysteine-type endopeptidase inhibitor, was significantly enriched, which were extensively characterized in SMPs^11,32^. It has been suggested that cysteine-type endopeptidase inhibitor might inhibit cysteine-type endopeptidase to degrade shell matrix proteins^43,44^. Our studies also revealed several enriched pathways that have been implicated in biomineralization, including ECM-receptor interaction, other types of O-glycan biosynthesis and ubiquitin mediated proteolysis^31,32,44^. Thus, a close relationship between the differentially expressed lncRNAs and biomineralization was observed.

Although pigmentation is a multifactorial phenotypic traits, only a small numbers of pathways regulating pigmentation have been validated to date^9^. Of that, Jak-STAT signaling pathway^34,35^, Endocytosis^20,36^, and Notch signaling pathway^20,22^ have been identified in our study. It is worth noting that several pigment biosynthesis related terms, such as tryptophan metabolism and porphyrin and chlorophy2 metabolism were identified in the GO analysis. Tryptophan is used to synthesize ommochrome and substitute for tyrosine as an oxidizable substrate for melanin^16,45,46^. Porphyrin, a tetrapyrroles, was one of the shell pigments found in Mollusca. Therefore, data from our functional enrichment analysis suggest a close correlation between the differentially expressed lncRNAs and pigmentation.

Comparison of two RNA-seq datasets identified 349 protein-coding transcripts that were shared between DET and DEM. These mRNAs could be used as auxiliary materials to further investigate the pigmentation associated lncRNAs. A total of 6 mRNAs are selected to function in pigments biosynthesis involving in melanin, tetrapyrrole, carotenoid and ommochrome (Table 2). Melanin is the end-product of complex multistep transformation of tyrosine^15^, extensively existing in the organism kingdom. Tyrosinase is the key enzyme in pigment synthesis, initiating a cascade of reactions converting tyrosine to the melanin biopolymer^47^. In insects, multiple enzymes are identified to directly involve in melanogenesis including peroxidase, phenoloxidase (PO), dopachrome conversion enzyme (DCE)^26,48^. Furthermore, two types of insect POs have been identified in some insect species, that can be identified as tyrosinase-like and laccase-type^26^. In cephalopod, the melanin-producing pathway in the ink gland includes three main enzymes of tyrosinases, peroxidase and dopachrome rearranging enzymes^49^. Porphyrins, termed as cyclic structure tetrapyrroles, are found in bacteria, plants, and animals and are synthesized via the haem pathway11. Carotenoids can be transformed to apocarotenoids such as retinoids, whose metabolic were reported to be mediated by Cytochrome P450s^50–53^. Kynurenine 3-monooxygenase catalyses the hydroxylation of kynurenine to 3-hydroxykynurenine, which has a key role in tryptophan catabolism and synthesis of ommochrome pigments^54,55^.

Several studies indicate that the intricate mechanisms of pigmentation require a coordinated posttranscriptional regulatory network of genes expression. However, our knowledge on the role of lncRNAs in pigmentation is very limited^40,56^. Our studies demonstrated that chorion peroxidase and its *cis*-acting lincRNA TCONS_00951105 showed the highest expression level in black shell oyster. Chorion peroxidase was initially identified from *Drosophila melagaster* and reported to relate to eggshell chorion harden involving protein crosslinking and melanization in insects^57,58^. Moreover, higher levels of expression were found in left black mantle relative to right white mantle of oysters with asymmetric pattern of shell colour. Peroxidase has been suggested to serve in an alternative melanogenic pathway in insect and cephalopod. Peroxidase is associated with melanosomes in the ink gland, where it is thought to catalyze the formation of eumelanin^49,59^. Peroxidase has been identified in many DGE datasets^20,24^, strongly suggesting its role in shell pigmentation. Phylogenetic tree using 26 peroxidase in *C. gigas* showed the chorion peroxidase LOC105324712 (CGI 10011763) clustered with peroxidases in insect and cephalopod, which have been implicated in melanin biosynthesis^33^. Altogether, our studies suggest that chorion peroxidase and its *cis*-acting lincRNA TCONS_00951105 may play an important role in melanin synthesis and shell colour regulation (Fig. 3).

## Conclusion

This study provided a catalog of lncRNAs in mantle of five-month-old Pacific oysters and profiled their expression in four shell colours variants. We identified a total of 12,443 lncRNAs, encoded by 11,637 gene loci, consisting of 8,226 lincRNAs, 387 antisense lncRNAs, and 3,630 intronic lncRNAs. LncRNA transcripts showed a relatively short length with fewer exons and low expression relative to their counterpart protein coding RNA (mRNA) transcripts. We identified 427 lncRNA transcripts that are differentially expressed among six pairwise groups based on one replicate per sib family. Functional enrichment of differentially expressed lncRNA and mRNA transcripts showed that they are potentially associated with biomineralization and shell pigmentation. And a total of 6 mRNAs are identified to influence pigment biosynthesis including melanin, carotenoid, tetrapyrrole, and ommochrome. Finally, we selectively analyzed lncRNAs and target gene pairs, in which the lncRNAs and their target genes were differentially expressed between any two shell colours variants. Chorion peroxidase, a pigmentation associated gene, was found to be the *cis*-acting target of lincRNA (TCONS_00951105) simultaneously. Collectively, our studies of *C. gigas* mantle lncRNAs and their association with pigmentation might facilitate the selection of elite oyster lines with desired coloration patterns.

## Electronic supplementary material

Supplementary Figure

Supplementary Table S1

Supplementary Table S2

Supplementary Table S3

Supplementary Table S4

Supplementary Table S5

Supplementary Table S6

Supplementary Table S7

Supplementary Table S8

Supplementary Table S9
