# Supplementary material for: Transcriptional profiling of long non-coding RNAs in mantle of Crassostrea gigas and their association with shell pigmentation
Source: Sci Rep. 2018 Jan 23;8:1436. doi: 10.1038/s41598-018-19950-6 (PMC5780484; doi:10.1038/s41598-018-19950-6)

Transcriptional profiling of long non-coding RNAs in mantle of *Crassostrea gigas* and their association with shell pigmentation

Dandan Feng[1] ·Qi Li[1,2] ·Hong Yu[1] ·Lingfeng Kong[1] ·Shaojun Du[3]

1 Key Laboratory of Mariculture, Ministry of Education, Ocean University of China, Qingdao 266003, China

2 Laboratory for Marine Fisheries Science and Food Production Processes, Qingdao National Laboratory for Marine Science and Technology, Qingdao 266237, China

3 Institute of Marine and Environmental Technology, Department of Biochemistry and Molecular Biology, University of Maryland School of Medicine, Baltimore, MD, United States

*corresponding author: qili66@ouc.edu.cn

Figure S1 TE components in three subtypes of oyster lncRNAs and mRNAs. The main TE families were identified using RepeatMasker. Percentage of TE components in mRNAs and individual subtype of lncRNAs were represented.

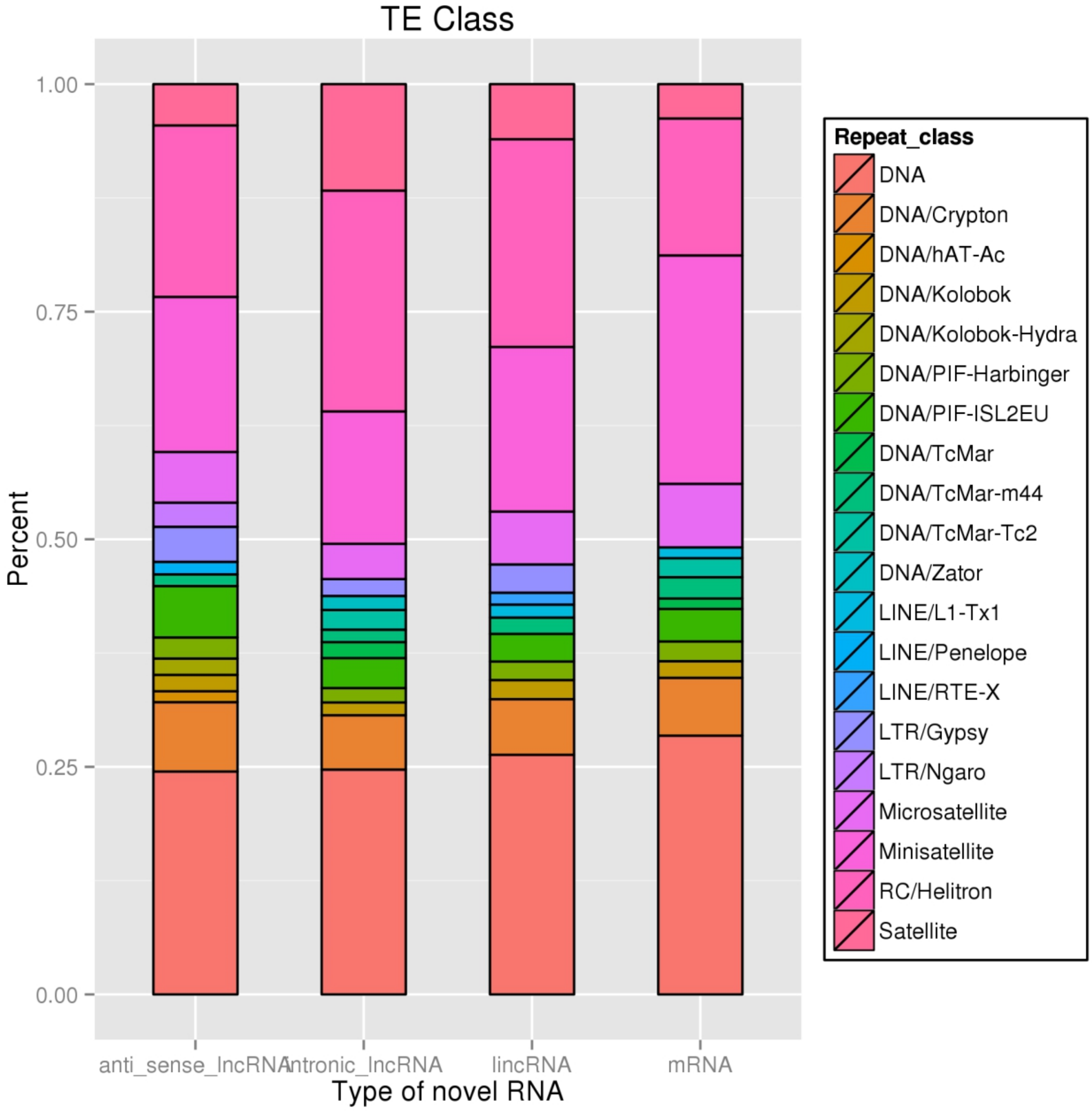

Figure S2 Cluster analysis of differentially expressed lncRNA and mRNA transcripts. a. Cluster analysis of differentially expressed lncRNA transcripts. b. Cluster analysis of differentially expressed mRNA transcripts.

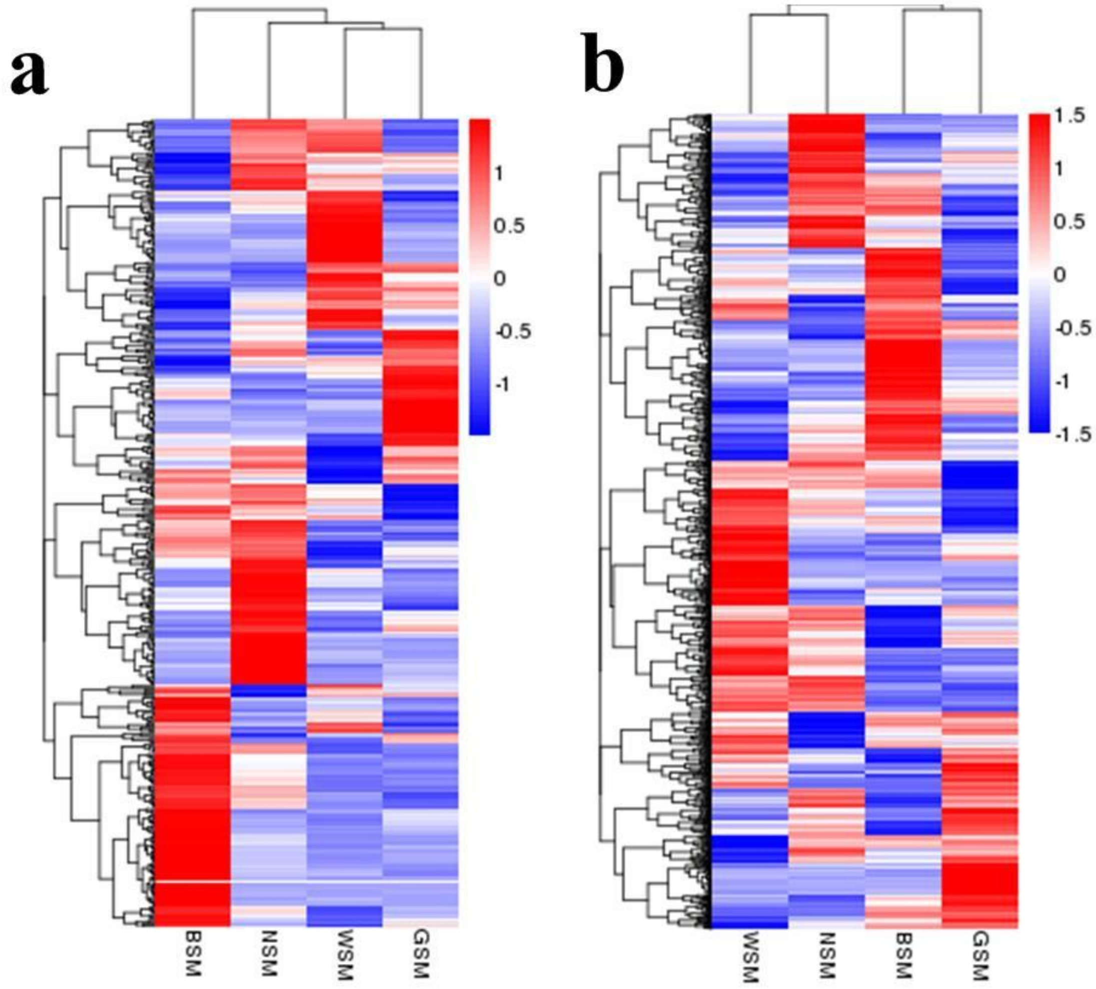

Supplement: Supplementary file 1 — Supplementary Figure [file 41598_2018_19950_MOESM1_ESM.pdf]
